# LDRGDb - Legumes disease resistance genes database

**DOI:** 10.3389/fpls.2023.1143111

**Published:** 2023-04-18

**Authors:** Harshita Saxena, Aishani Kulshreshtha, Avinav Agarwal, Anuj Kumar, Nisha Singh, Chakresh Kumar Jain

**Affiliations:** ^1^ Department of Biotechnology, Jaypee Institute of Information Technology, Noida, India; ^2^ Department of Microbiology and Immunology, Dalhousie University, Halifax, NS, Canada; ^3^ Department of Bioinformatics, Gujarat Biotechnology University, Gandhinagar, India

**Keywords:** disease resistance genes, genomics, LDRGDb, legumes, proteomics, QTLs data

## Abstract

Legumes comprise one of the world’s largest, most diverse, and economically important plant families, known for their nutritional and medicinal benefits. Legumes are susceptible to a wide range of diseases, similar to other agricultural crops. Diseases have a considerable impact on the production of legume crop species, resulting in large yield losses worldwide. Due to continuous interactions between plants and their pathogens in the environment and the evolution of new pathogens under high selection pressure; disease resistant genes emerge in plant cultivars in the field against those pathogens or disease. Thus, disease resistant genes play critical roles in plant resistance responses, and their discovery and subsequent use in breeding programmes aid in reducing yield loss. The genomic era, with its high-throughput and low-cost genomic tools, has revolutionised our understanding of the complex interactions between legumes and pathogens, resulting in the identification of several critical participants in both the resistant and susceptible relationships. However, a substantial amount of existing information about numerous legume species has been disseminated as text or is preserved across fractions in different databases, posing a challenge for researchers. As a result, the range, scope, and complexity of these resources pose challenges to those who manage and use them. Therefore, there is an urgent need to develop tools and a single conjugate database to manage genetic information for the world’s plant genetic resources, allowing for the rapid incorporation of essential resistance genes into breeding strategies. Here, developed the first comprehensive database of disease resistance genes named as LDRGDb - LEGUMES DISEASE RESISTANCE GENES DATABASE comprises 10 legumes [Pigeon pea (*Cajanus cajan*), Chickpea (*Cicer arietinum*), Soybean (*Glycine max*), Lentil (*Lens culinaris*), Alfalfa (*Medicago sativa*), Barrelclover (*Medicago truncatula*), Common bean (*Phaseolus vulgaris*), Pea (*Pisum sativum*),Faba bean (*Vicia faba*), and Cowpea (*Vigna unguiculata*)]. The LDRGDb is a user-friendly database developed by integrating a variety of tools and software that combine knowledge about resistant genes, QTLs, and their loci, with proteomics, pathway interactions, and genomics (https://ldrgdb.in/).

## Introduction

Legumes are seed plants belonging to the Leguminosae family, spanning more than 13000 species across 600 genera. Legumes are diverse; they are suitable for cultivation in a variety of environments and temperatures (Magrini et al., 2019). They are an excellent source of antioxidants, micronutrients, and proteins, finding use as natural fertilizers, medicines, and animal fodder (Shah et al., 2020; Singh et al., 2022). Apart from their health benefits, legumes naturally have the ability to fix nitrogen from the atmosphere symbiotically, which has positive effects on agriculture and soil enrichment that are both long-lasting and economically viable. Improvement in legume cultivation and their increased usage can help ensure food security and contribute to better soil fertility (Foyer et al., 2016; Singh et al., 2020).

**Figure 1:**
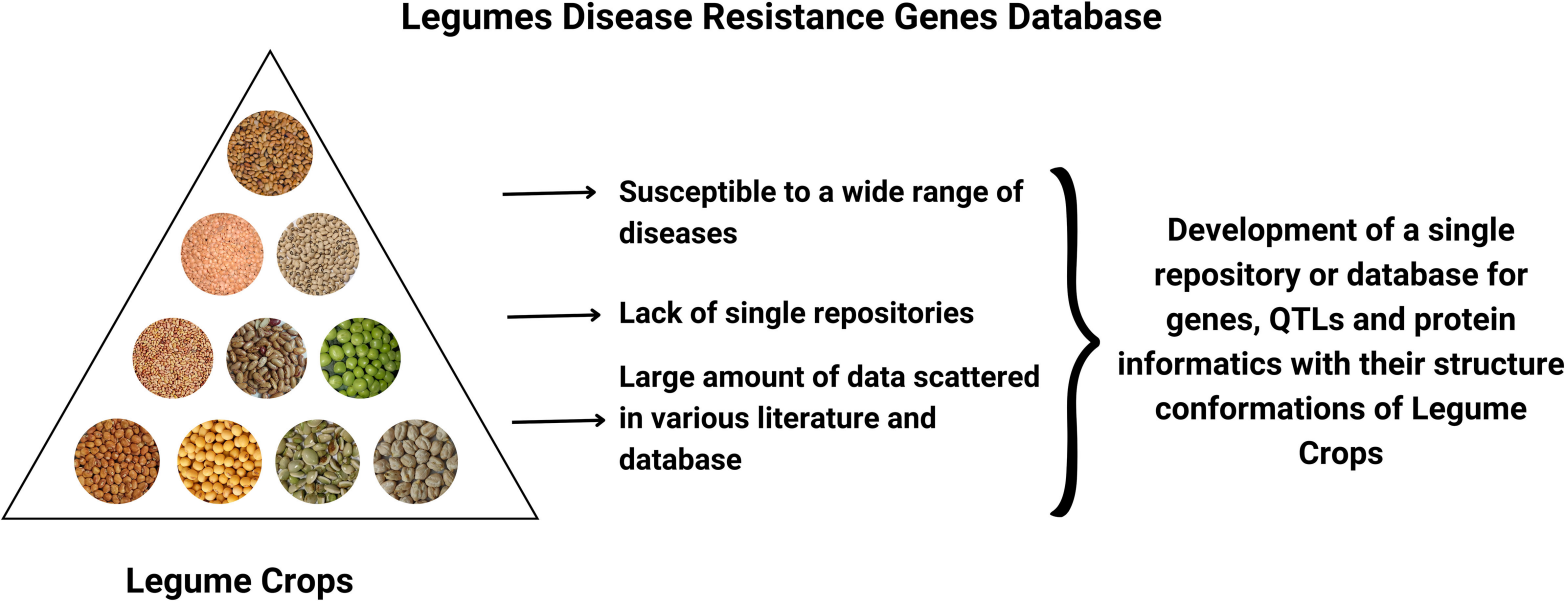
Graphical Abstract.

However, one of the main impediments in the way of abundant and quality yield of legume crops is pests and diseases **Figure 1**. Furthermore, climate changes have made it easier for various species to move freely, which has led to the emergence of new diseases that could grow into uncontrollable epidemics and endanger food security (Piquerez et al., 2014). Thus, crop improvement is a key component of sustainable agriculture and can be accomplished using a variety of techniques, ranging from traditional breeding to genetic engineering. To increase the effectiveness of the breeding process, it is necessary to have a thorough understanding of host-pathogen interactions as well as effective resistance mechanisms at the cellular, genetic, and molecular levels. The most effective, affordable, and environmentally friendly of the various control techniques available is breeding for resistance (Rubiales et al., 2014). These factors drive plant breeders and scientists to focus their efforts on finding mechanisms for plant disease resistance (Osuna-Cruz et al., 2018). Indeed, studies into the genes and genomes of legumes have provided valuable insight into disease resistance components and other agronomic traits (Leal-Bertioli et al., 2009; Khera et al., 2018; Zhuang et al., 2019; Sampaio et al., 2020).

Resistance genes (R genes) are variable plant genes that confer resistance to vast varieties of biotrophic pathogens, including viruses. As they can specifically recognise the matching pathogen effectors or associated protein(s), resistance (R) genes are the most potent defences against pathogen invasion (Liu et al., 2007). This allows plants to mount an effective defence at the site of infection. R genes are capable of expressing PR proteins in response to physical or chemical stimuli (Rodríguez-Sifuentes et al., 2020). As PR proteins have hydrolytic activity, contact toxicity, involvement in defence signalling, and pathogen enzyme inhibition, they constitute a variety of weapons against pathogens. We have seen that plant R genes have been divided into several groups based on their typical domains (Zheng et al., 2016). A class of proteins containing nucleotide binding (NB) and leucine-rich repeat (LRR) domains is encoded by the majority of R genes (Friedman and Baker, 2007).

**Figure 2:**
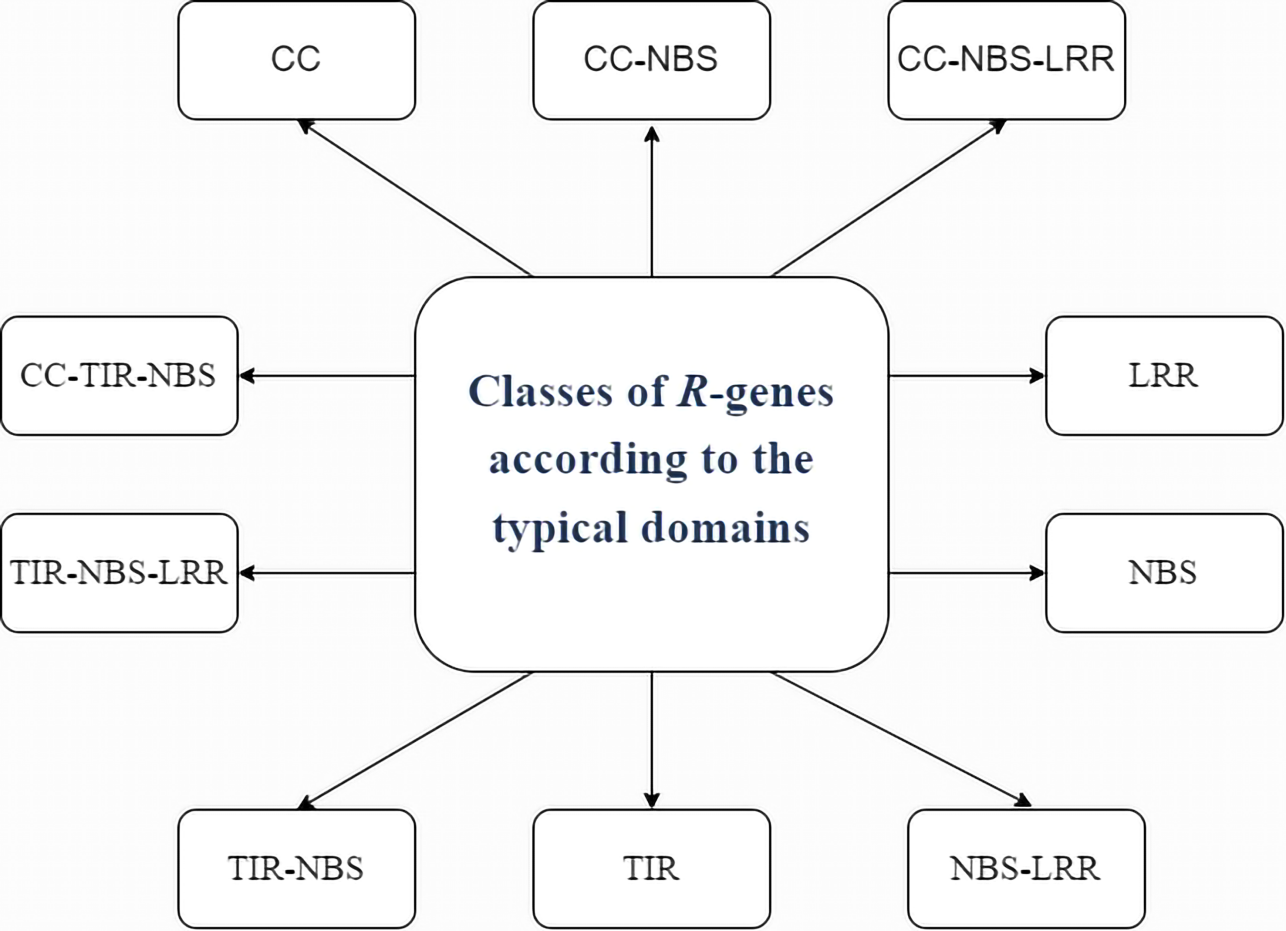
Image depicting various classes of R-Gene.

It is now known that R genes also produce proteins with a series of carboxy-terminal leucine-rich repeats (LRRs), a putative amino-terminal signalling domain, and a nucleotide binding site (NBS). There are two primary classes of “NBS-LRR” proteins: first which encodes an amino-terminal coiled-coiled motif (CC-NBS-LRR or CNL proteins) and second which has an amino-terminal TIR (Toll/interleukin receptor) domain (which are known as TIR-NBS-LRR or TNL proteins) (Meyers et al., 2005). **Figure 2** depicts the various classes of R-Gene. Hence, the participation of multiple R genes, post-transcriptional regulators, and biotic and abiotic stressors limits the ability of plants to resist disease.

R gene mechanisms work on the gene-for-gene concept (Kaloshian, 2004). The resistance occurs only when the R gene proteins engage with the pathogen Avr gene in a certain form. They can interact with the pathogen gene in two ways, first by directly interacting with its protein product and second, if it plays a catalytic role by interacting with something created by the Avr gene product (Hulbert et al., 2001). Any attempt of an infection is thus governed by both the genotype of the host and that of the pathogen. Once this recognition has taken place, the defence reactions are triggered. Hypersensitive reaction is frequently characterized in these defence reactions, which results in the death of the initial cell or cells infected and also the local accumulation of antimicrobial compounds (Moffett, 2009). Phytophthora root and stem rot in nonhost common bean has been found to induce a strong hypersensitive response to *Phytophthora sojae* due to upregulation of genes promoting phaseollidin and glyceollin production, which have significant inhibitory effects on oospore production and mycelial formation (Bi et al., 2022). Transcription factors have also been implicated in multilayer defence signalling against *Fusarium* wilt disease in chickpea (Chakraborty et al., 2020). Legumes exhibit resistance through other means as well; a recent study on Faba beans indicated that its resistant genotype withstood the Chocolate spot infection caused by *Botrytis fabae* due to a better Photosystem II Repair mechanism at early stages of the infection (Castillejo et al., 2021). Leaf spot infected alfalfa, when colonized by arbuscular mycorrhizal fungus (AMF), has shown to ameliorate the effects of the infection, thus displaying the potential to serve as a biocontrol strategy for leaf spot infection of alfalfa (Li et al., 2019).

Development of cost-effective next generation sequencing platforms with enhanced performances have given way to copious amounts of data. In order for these data to be effectively analysed and compared, various data management practices are being employed (Bauchet et al., 2019). Many databases exist for comprehensive study and management of the insurmountable data generated by plant genome studies. However, these repositories either only focus individually on model genomes such as soybean (https://soybase.org/) (Grant et al., 2009), *Medicago truncatula* (https://www.legoo.org) (Carrere et al., 2019), (http://www.medicago.org/MtDB) (Lamblin, 2003), or a combination of multiple model species (http://www.plantgdb.org/) (Dong, 2004), (http://plantgrn.noble.org/LegumeIP/) (Li et al., 2011). Currently, there is a lack of repositories that can pool the numerous disease resistance genes with proteomics and facilitate the quick integration of crucial resistance genes into breeding methods. Keeping this in mind, we have established and developed the first comprehensive database of disease resistance genes in legume plants named as LDRGDb, that incorporates knowledge about resistant genes, QTLs, and their loci, with proteomics, pathways interactions as well as structural conformations of proteins. The database serves as a medium for comprehensive search and retrieval of relevant information, and will help researchers understand various biological phenomena with relative ease. Our database has been constructed so as to aid in research of disease resistant common legume cultivars and their underlying mechanisms. LDRGDb spans 10 legume species, with genes, QTL information such as linkage group, neighbouring marker, population, maternal and paternal parent, proteomics, informatics such as molecular weight, theoretical pI, length of amino acids, half-life of protein, and aliphatic index with emphasis on structural conformation, which can aid in a thorough understanding of various pathways and interactions involved in disease resistance, functioning as a single repository for assisting in research.

## Methodology

### Data collection

**Figure 3:**
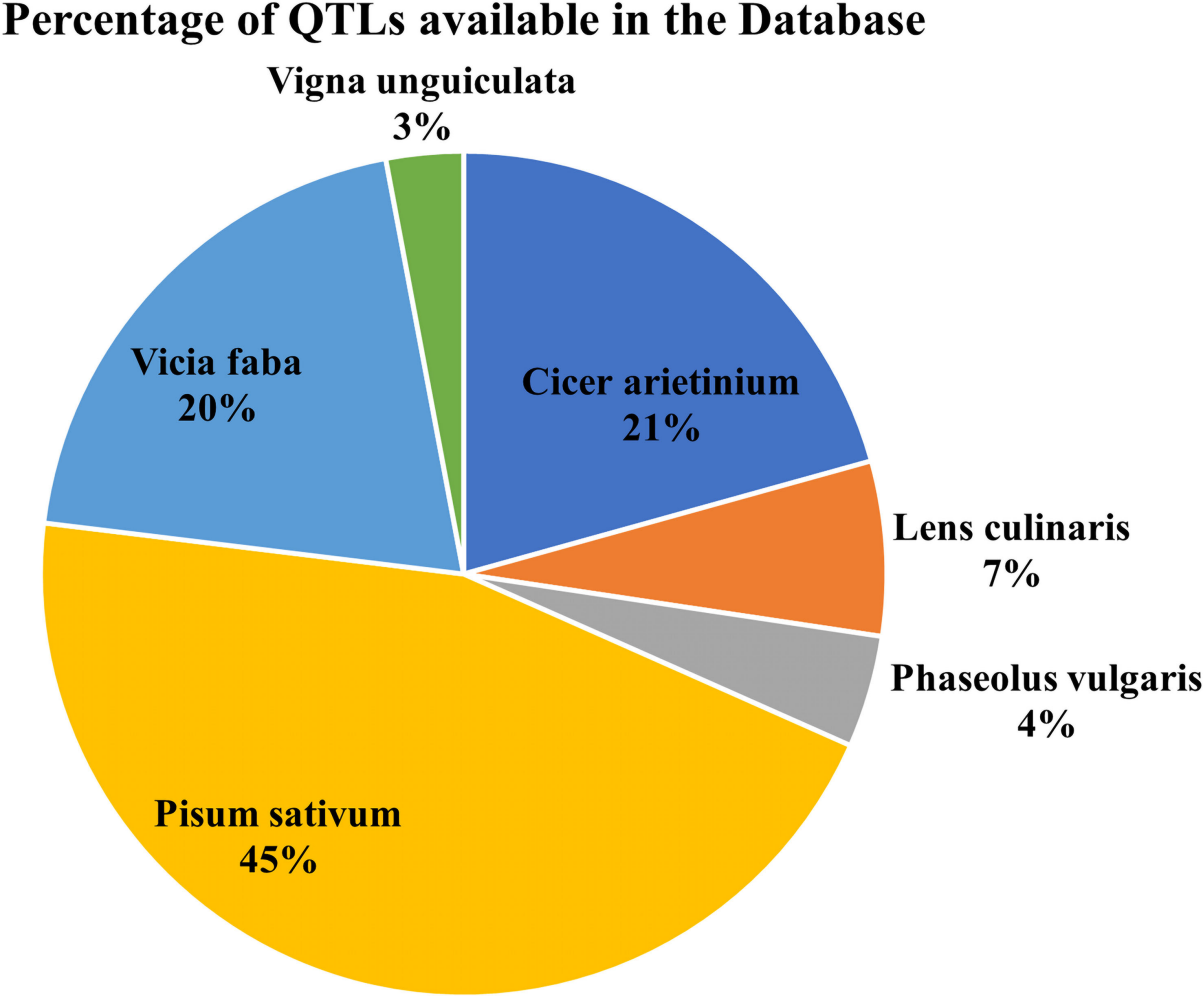
Percentage of QTls available in the Database.

**Figure 4:**
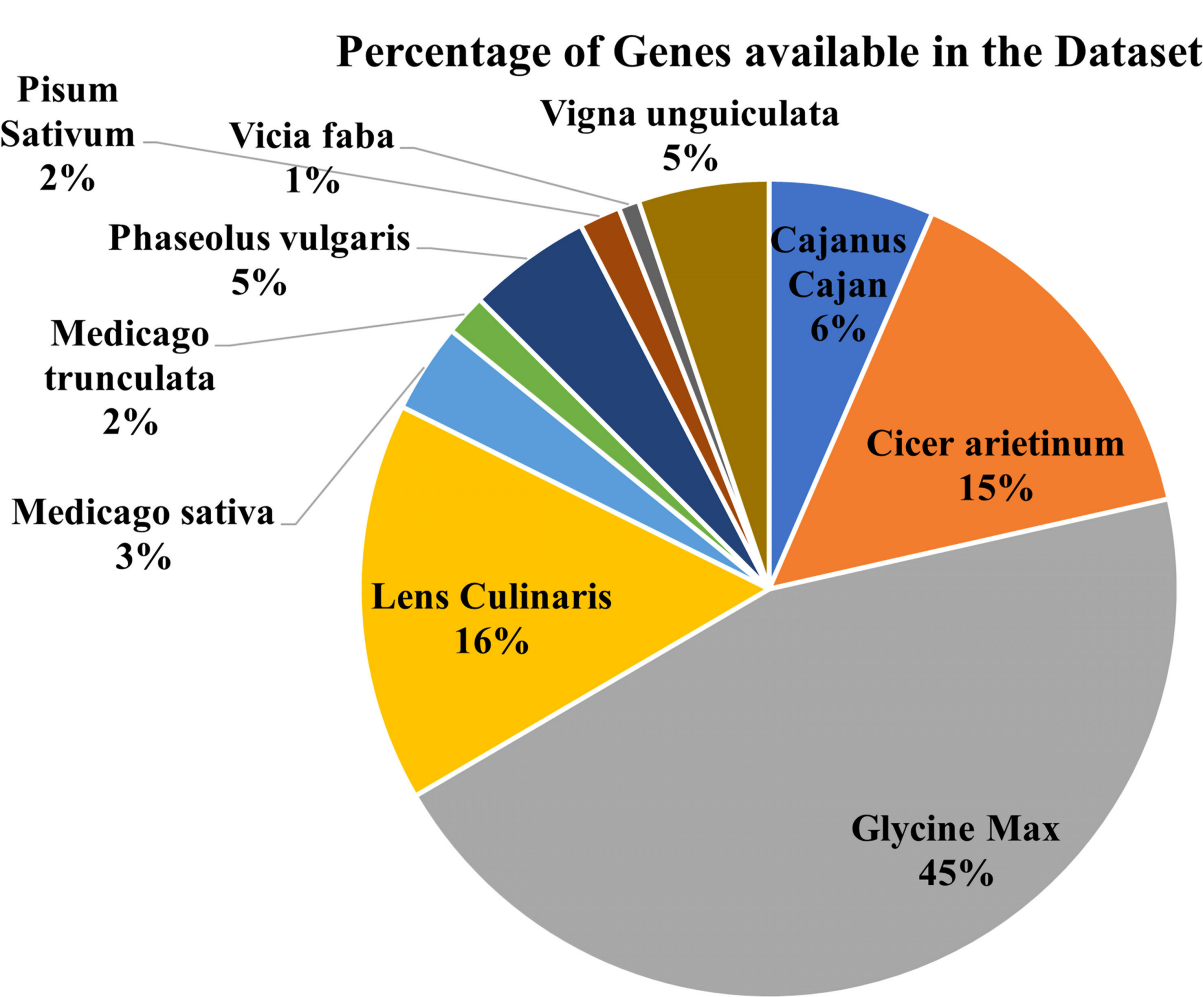
Percentage of Genes available in the Database.

The database has major 10 legume crops with their common disease-related genes and QTLs incorporated. QTLs data has been combined from numerous databases for a single model organism, such as soybase (Grant et al., 2009), databases for several species, such as pulsedb (Humann et al., 2019), prgdb (Calle García et al., 2021), and other databases. Further, the QTLs related to diseases were manually curated from the available scientific literature, books and journals. We incorporated gene data from various journals and books using the Pubmed (PubMed Labs, 2021) search box and varied search queries incorporating different diseases and crops from the last 5 years. (**Figures 3**, **4**) show the number of genes and QTLs incorporated into the database.

### Annotation of genes and QTLs

We performed functional annotation studies at genes and QTLs levels to develop comprehensive information for the collected data. Uniprot (Bateman et al., 2020) was used to retrieve protein-related data of the genes while protparam (Gasteiger et al., 2005) was used to retrieve individual protein details such as theoretical pI, the total number of negatively and positively charged residues, protein’s half-life, aliphatic index, and molecular weight etc. To mine and integrate the biological, molecular, and cellular processes involved in genes Uniport was utilized, (Bateman et al., 2020) whereas Interpro (Paysan-Lafosse et al., 2022) database for protein family and domain retrieval. The Swiss model (Waterhouse et al., 2018) was used to incorporate the structure of the protein of a gene with its template included.

### Database design

**Figure 5:**
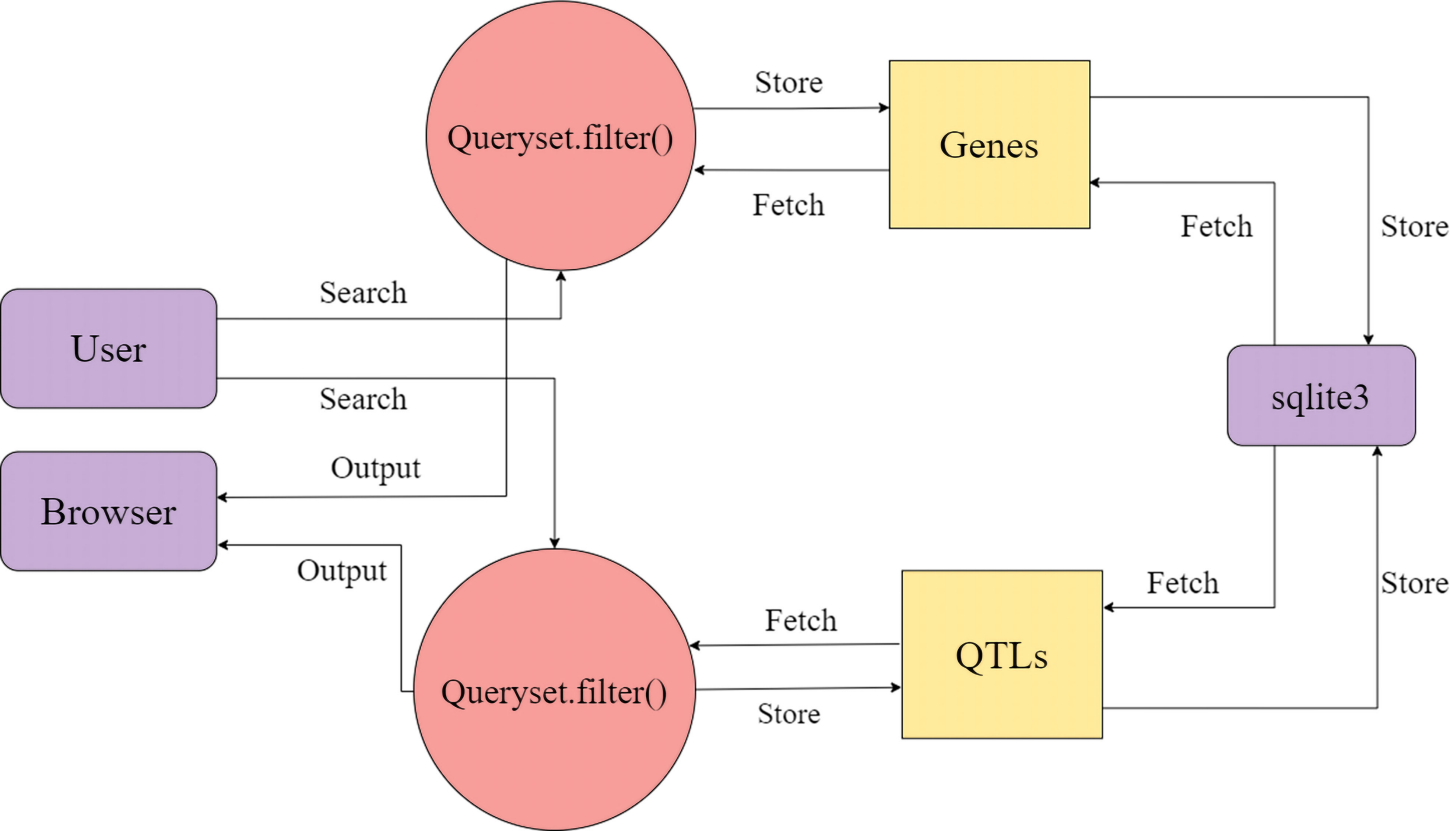
Depicting the data flow diagram of the program.

The database is made up of two tables: Genes and QTLs, and one-to-many relationships were utilised to create the complete database structure, allowing for the inclusion of any number of connections. The data’s detailed data flow is documented in (**Figure 5**). For website development, Django v4.1.3 with in-built sqlite3 has been utilized. Django offers speedy development, fast processing, and scalability for website development, while sqlite3 provides a lightweight disk-based database that doesn’t require a separate server process and allows accessing the database using a nonstandard variant of the SQL query language. Special care has been taken to protect the database’s structure, consistency, and the accuracy of the data stored.

### Key features

**Figure 6:**
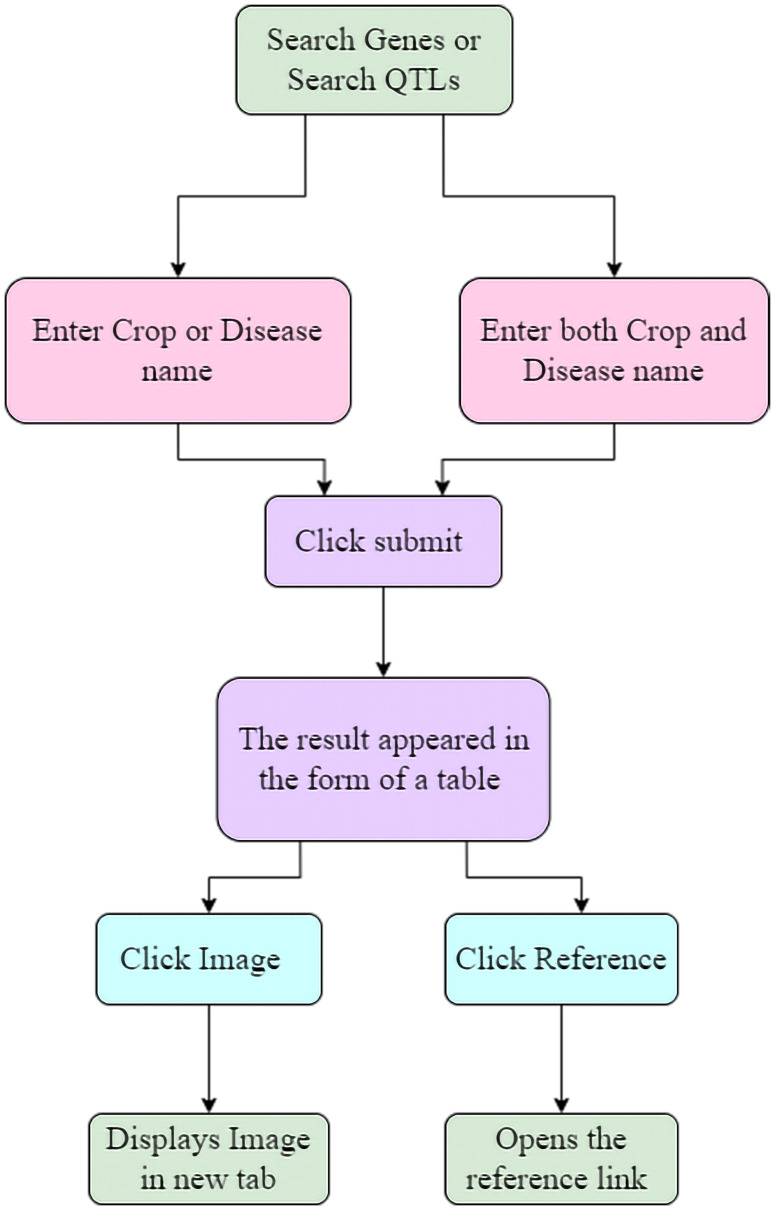
Depicting how to utilize the LDRGDb.

The LDRGDb is a comprehensive search and retrieval database of significant information that serves as a centralised repository for research purposes. It is the only legume repository that includes not only QTL information such as linkage group, neighbouring marker, population, and maternal and paternal parent, but also genes with proteomics information such as theoretical pI, the total number of negatively and positively charged residues, protein half-life, aliphatic index, and molecular weight, among other things. The inclusion of protein pathway interactions with diverse biological, cellular, and molecular processes aid researchers in understanding a wide range of processes. The incorporation of protein structural conformations with the template annotated through the Swiss model is a major aspect of the LDRGDb that can aid in full knowledge of protein interactions involved in disease resistance. (**Figure 6**) depicts how the LDRGDb is utilized for the retrieval of significant information.

## Conclusion

**Figure 7:**
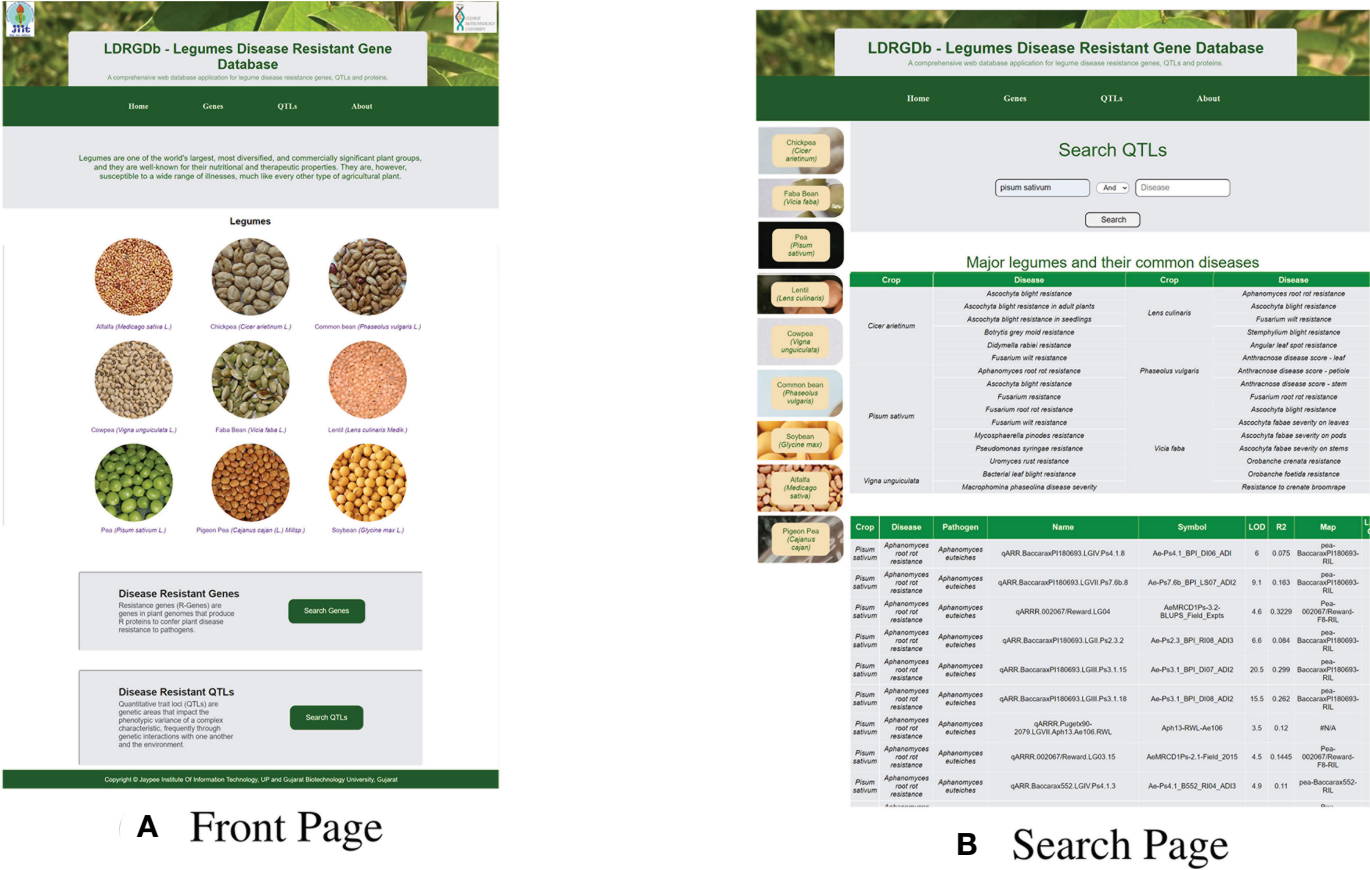
Images showing the **(A)** Front Page and **(B)** Search Page of the website.

Disease-resistant mechanisms of plants are constrained by the involvement of various genes, post-transcriptional controls, biotic and abiotic stresses. The understanding of these mechanisms and their impact on plant proteomes and metabolomes is important for improvement of legume production. To obtain knowledge about the mechanisms of resistance, LDRGDb serves as an exemplary one stop database for quick access of disease resistance genes, QTLs, and the proteins and pathways associated with them for various legume species. Users can search using either disease names, crop names, or both, facilitating easy and comprehensive ingress into the data. The data collected from various databases and literature has been meticulously structured to allow for rapid searches for diseases, legumes and the genes associated with them using user-friendly web interfaces. The database has specific sections for particular crop characteristics, prevalent diseases, as well as a FAQ section for frequently asked questions. These are the webpage screenshots (**Figure 7**).

## Data availability statement

The original contributions presented in the study are included in the article/supplementary material. Further inquiries can be directed to the corresponding author.

## Author contributions

HS contributed to collection of data implementation and initial draft writing. AiK contributed to collection of data, initial draft writing tables, and figures. AA contributed to collection of data and initial draft writing. AK contributed to designing and editing the manuscript. NS conceived, supervised and edited the final manuscript. CJ conceived, supervised and edited the final manuscript. All authors contributed to the article and approved the submitted version.
